# The effect of different surface treatments of demineralised enamel on microleakage under metal orthodontic brackets

**DOI:** 10.1186/2196-1042-14-2

**Published:** 2013-05-20

**Authors:** Horieh Moosavi, Farzaneh Ahrari, Hamideh Mohamadipour

**Affiliations:** 1 Dental Material Research Center, School of Dentistry, Mashhad University of Medical Sciences, Mashhad, Iran; 2 Dental Research Center, School of Dentistry, Mashhad University of Medical Sciences, Mashhad, Iran; 3 School of Dentistry, Mashhad University of Medical Sciences, Mashhad, Iran

## Abstract

**Background:**

The aim of this investigation was to assess the effects of different treatments of demineralised enamel on microleakage under orthodontic brackets.

**Methods:**

Seventy-five intact premolars were randomly assigned to five groups. The teeth in groups 2 through 5 were immersed in a demineralising solution for 16 weeks. In groups 1 (control) and 2 (demineralised/control), conventional acid etching was used. In group 3, sodium hypochlorite (NaOCl) was applied on the enamel surface for 1 min after acid etching, and in group 4, Transbond Plus (3M Unitek, Monrovia, CA, USA) self-etching primer (SEP) was used. The teeth in group 5 were treated with 2% sodium fluoride (NaF) for 4 min before etching. After bracket bonding, the specimens were thermocycled, sealed with nail varnish, immersed in 0.5% basic fuchsine solution for 24 h and sectioned. Microleakage was measured under a stereomicroscope for the enamel-adhesive and adhesive-bracket interfaces of both occlusal and gingival sides.

**Results:**

Demineralised teeth showed more microleakage at the enamel-adhesive interface on both occlusal and gingival sides compared to sound teeth, but the difference was not significant (*P* > 0.005). Treating the demineralised enamel with 5% NaOCl or Transbond Plus SEP was not effective in reducing microleakage. NaF treatment followed by acid etching of demineralised enamel resulted in significantly lower microleakage in most comparisons (*P* < 0.005).

**Conclusions:**

The use of 2% NaF on hypomineralised enamel before the bracket bonding procedure is an effective way to decrease microleakage.

## Background

In contemporary orthodontic treatments, resin composites are widely used for bracket bonding. One of the major disadvantages of these materials is polymerisation shrinkage, which may cause leakage between the tooth-adhesive or adhesive-bracket interfaces, resulting in the penetration of bacteria and fluids in these areas. Microleakage beneath orthodontic brackets can have severe consequences such as enamel decalcification, enamel discoloration, corrosion and decreased bond strength. Enamel decalcification or the development of white spot lesions is a great concern for patients undergoing fixed orthodontic treatment. Previous studies indicated a significantly high prevalence of white spot lesions during orthodontic treatment [1,2] that may occur in as early as 4 weeks with inadequate oral hygiene [3]. Although James et al. [4] found no correlation between microleakage and bond strength, several authors believed that there is a relationship between the adequacy of adhesion and microleakage resistance [5,6].

A significant number of patients seeking orthodontic treatment have local or generalised hypomineralised areas in one or more teeth due to hereditary or environmental factors. Enamel hypomineralisation may be a result of incipient caries or may be due to a systemic condition known as molar incisor hypomineralisation (MIH). MIH has been defined as a hypomineralisation of systemic origin in one or more first permanent molars which are frequently associated with affected incisors [7]. A severe form of enamel hypomineralisation is also observed in hypocalcified and hypomaturation types of amelogenesis imperfecta (AI) [8], a congenital disease that affects the entire dentition. From a clinical point of view, it may be possible that the decreased adhesion between the adhesive and defective hypomineralised enamel results in a remarkable amount of microleakage under orthodontic brackets bonded to this type of enamel.

A few studies evaluated the adhesion of adhesive resins to hypomineralised enamel and suggested some methods to improve the bonding interface. Pretreatment of hypomineralised enamel with 5% sodium hypochlorite (NaOCl) has been recommended to remove excess enamel proteins (deproteinisation), thus improving the bond strength [9-12]. Some authors [13] believe that self-etching adhesives bond better to hypomineralised enamel than total-etch systems. It has also been revealed that fluoride treatment before acid etching of enamel caries or hypomineralised enamel can restore the mineral lost during lesion formation while providing etching patterns that are suitable for composite placement [14-16].

Previous studies of microleakage under orthodontic brackets evaluated the effects of light curing [4,17,18], type of adhesive [6,19-21] and method of enamel preparation [22,23], but, to our knowledge, no study has investigated microleakage under orthodontic brackets bonded to hypomineralised enamel. The aim of this study was to compare the effects of several surface treatments including 5% NaOCl, Transbond Plus self-etching primer and 2% sodium fluoride (NaF) on microleakage at the enamel-adhesive-bracket complex for the occlusal and gingival margins of brackets bonded to demineralised enamel.

## Methods

Seventy-five maxillary premolar teeth without caries, cracks and developmental defects were used in this study. The teeth were cleaned with a scaler to remove soft tissue remnants and callus and were stored in a 0.1% thymol solution to inhibit bacterial growth until the time of the experiment. The sample was randomly divided into five groups of 15 teeth each. The first group served as the control, whereas the teeth in other groups were immersed for 16 weeks in a cariogenic solution to produce demineralised enamel. This solution consisted of 2.2 mM CaCl_2_, 2.2 mM NaH_2_PO_4_ and 50 mM acetic acid, with pH adjusted to 4.8 using KOH [24]. The specimens were immersed individually in plastic containers with approximately 10 ml of cariogenic solution, and the solution was replaced weekly.

Before bonding, the enamel surface was polished with non-fluoridated pumice slurry and rubber cups for 5 s, rinsed with water and air-dried. Stainless steel standard edgewise second premolar brackets (0.018-in slot; Dentaurum, Ispringen, Germany) were used in this study. The teeth were prepared for bracket bonding according to one of the following surface treatment procedures:

•*Group 1 (control)*. The enamel surfaces of intact teeth were etched with 37% phosphoric acid gel (Ortho Organizers Inc., San Marcos, CA, USA) for 30 s, then rinsed with water for 15 s and dried with an oil-free air source for 10 s. A thin layer of Transbond XT primer (3M Unitek, Monrovia, CA, USA) was later applied on the etched surface, and the bracket was placed at the middle of the clinical crown using Transbond XT adhesive (3M Unitek). The flash material was removed from around the base with a dental explorer, and the adhesive was cured for 10 s from each of the occlusal, gingival, mesial and distal directions (40 s in total) using a light-emitting diode device (Bluephase C8; Ivoclar Vivadent, Schaan, Liechtenstein) at a power density of 650 mW/cm^2^.

•*Group 2 (demineralised/control)*. The bonding procedure was the same as the control group (Group 1), but brackets were bonded on hypomineralised enamel.

•*Group 3 (demineralised/NaOCl)*. After acid conditioning, a 5% sodium hypochlorite solution was applied on the enamel surface for 1 min and then rinsed and dried. The subsequent steps were the same as the control group.

•*Group 4 (demineralised/SEP)*. Transbond Plus self-etching primer (3M Unitek) was activated according to the manufacturers' instructions and rubbed gently onto the enamel surface for 10 s, then thinned with a water- and oil-free air source for 1 to 2 s. Brackets were bonded with Transbond XT adhesive and light cured similarly to the control group.

•*Group 5 (demineralised/NaF)*. A 2% neutral sodium fluoride gel (Sultan Healthcare Inc., Englewood, NJ, USA) was applied on the enamel surface for 4 min. The teeth were then rinsed with water for two consecutive periods of 5 min each to remove any readily soluble reaction products [15], and then the teeth were etched with 37% phosphoric acid gel and bonded similar to the control group.

After bracket bonding, the teeth were stored in distilled water in plastic containers for 24 h at 37°C and then subjected to a thermocycling process. Thermocycling was performed between 5 ± 1°C to 55 ± 1°C for 500 cycles, with a dwell time of 30 s per bath.

### Microleakage evaluation

Dye penetration was used for microleakage assessment. At first, the teeth apices were sealed with sticky wax. The specimens were dried and then coated with two layers of nail varnish so that only 1 mm of the enamel beyond the bracket margins was exposed. The teeth were immersed in 0.5% basic fuchsine solution for 24 h at room temperature, then thoroughly rinsed with tap water and embedded in epoxy resin. Two parallel longitudinal sections were made in a buccolingual direction through the occlusal surface using a low-speed diamond saw (D&Z, Switzerland).

One calibrated examiner evaluated the sections under a stereomicroscope (Dino-Lite Pro, AnMo Electronics Corp, Taiwan) at ×50 magnification. Microleakage was determined by measuring the deepest dye penetration from the occlusal and gingival margins of the brackets at both the enamel-adhesive and adhesive-bracket interfaces using an electronic digital callipers; the data were recorded in a range from 0.0 to 5.0 mm. To examine the measurement error, 15 specimens were randomly selected and re-examined 2 weeks later.

### Statistical analysis

For each specimen, the microleakage values of the gingival and occlusal sides were achieved by calculating the mean microleakage of each side measured from two sections. Comparisons of the microleakage values were made individually for occlusal and gingival sides at each of the enamel-adhesive and adhesive-bracket interfaces. Statistical analysis was performed by SPSS (Statistical Package for the Social Sciences, Version 16, Chicago, IL, USA) software, using Kruskal-Wallis and Mann-Whitney *U* tests with Bonferroni correction. For each of the enamel-adhesive and adhesive-bracket interfaces, the measurement error was calculated using the Dahlberg formula (*s*^2^ = ∑ *d*^2^/2*n*), and the systemic error was determined by Wilcoxon signed-rank test. The level of significance was predetermined at *P* < 0.05.

## Results and discussion

### Results

The measurement error was calculated to be 0.059 mm for the enamel-adhesive and 0.026 mm for the adhesive-bracket interfaces. The intra-examiner systemic error was not significant between the two measurements (*P* = 0.119 for the enamel-adhesive and *P* = 0.593 for the adhesive-bracket interfaces).

**Figure 1:**
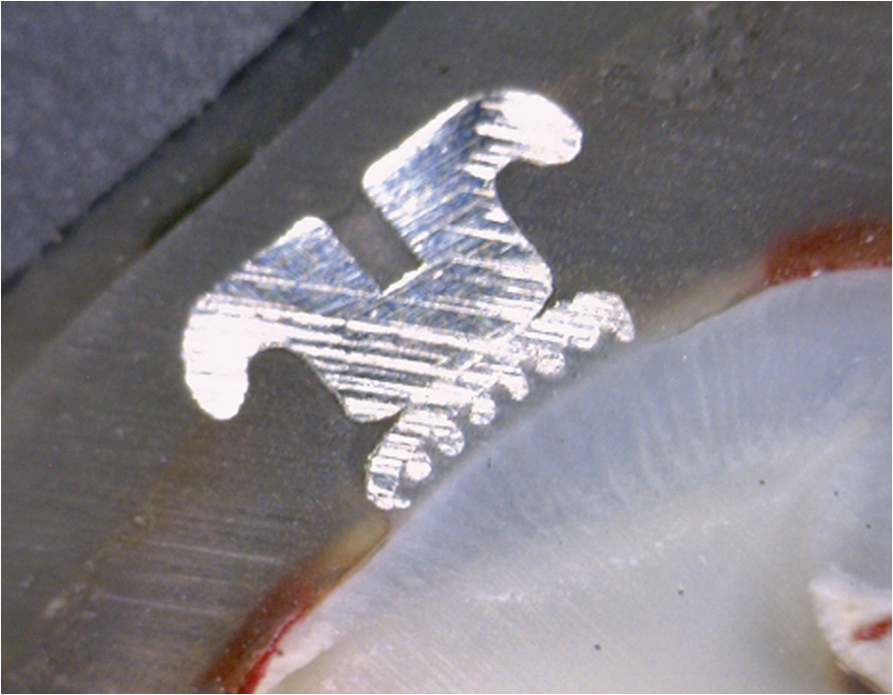
Microleakage was not seen under a metal bracket.

**Figure 2:**
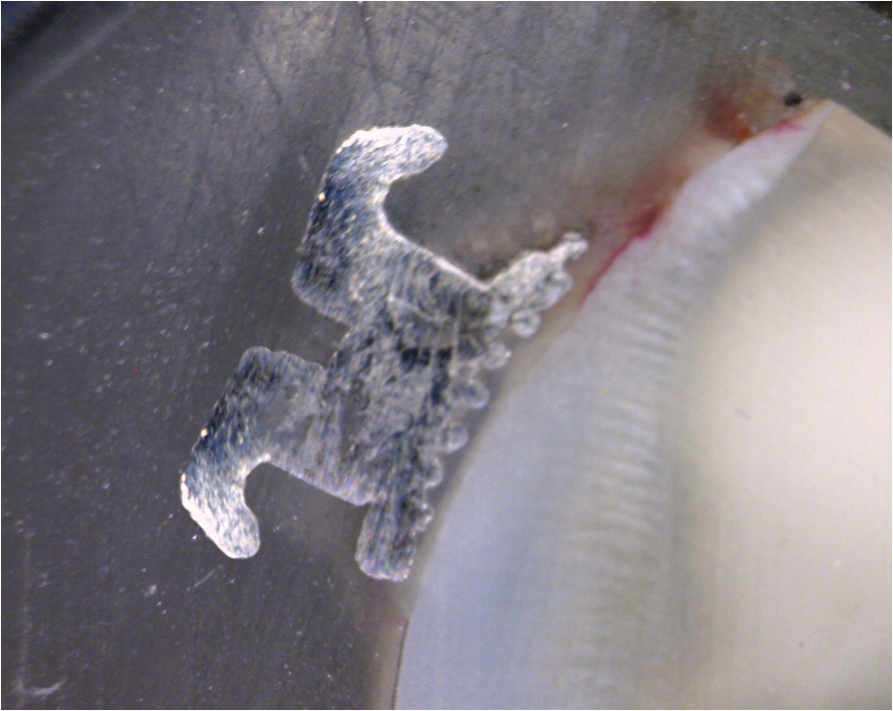
Microleakage at the enamel-adhesive interface.

Table 1 presents the descriptive statistics and comparisons of microleakage values between the study groups and between the occlusal and gingival sides of each group. No significant difference was found in any of the study groups between microleakage values of the occlusal and gingival sides, either at the enamel-adhesive or at the adhesive-bracket interfaces (*P* > 0.05; Table 1). The Kruskal-Wallis test exhibited significant differences in microleakage values of the occlusal and gingival sides at the enamel-adhesive and adhesive-bracket interfaces among the study groups (Table 1). Between group comparisons by Mann-Whitney *U* test revealed that at the enamel-adhesive interface, group 5 (demineralised/NaF) had a statistically lower amount of microleakage than the other study groups (*P* < 0.005; Table 1). Demineralised teeth prepared by conventional acid etching (group 2) revealed greater microleakage values at the enamel-adhesive interface on both the occlusal and gingival sides compared with those of the control group (group 1), but the difference was not statistically significant (*P* > 0.005). When the adhesive-bracket interface was considered, group 4 (demineralised/SEP) displayed significantly higher microleakage values than did groups 2 (demineralised/control) and 5 (demineralised/NaF) along both occlusal and gingival margins (*P* < 0.005; Table 1). Group 4 (demineralised/SEP) also revealed significantly more microleakage than group 3 (demineralised/NaOCl) at the adhesive-bracket interface of the gingival side (*P* < 0.005; Table 1). Pairwise comparisons between microleakage values of other groups were not statistically significant either at the enamel-adhesive or at the adhesive-bracket interfaces (*P* > 0.005). Figure 1 demonstrates no microleakage, while Figure 2 illustrates microleakage at the enamel-adhesive interface under the bracket.

**Table 1:** Descriptive statistics and comparison of microleakage values between the study groups for occlusal and gingival sides

**Interface**	**Group**	**Occlusal**				**Gingival**				**Statistical significance**
		**Descriptive values (mm)**			**Pairwise comparisons**	**Descriptive values (mm)**			**Pairwise comparisons**	
		**Percentiles**				**Percentiles**				
		**25th**	**50th (median)**	**75th**		**25th**	**50th (median)**	**75th**		
Enamel-adhesive	1 (Control)	0	0	0.39	a	0	0	0.84	a	*P* = 0.479
	2 (Demineralised/control)	0	0	1.39	a	0	0	1.99	a	*P* = 0.358
	3 (Demineralised/NaOCl)	0	0	1.27	a	0	0	1.34	a	*P* = 0.749
	4 (Demineralised/SEP)	0	0.31	0.95	a	0	0.72	1.24	a	*P* = 0.409
	5 (Demineralised/NaF)	0	0	0		0	0	0		*P* = 0.317
	Statistical significance	*P* = 0.007				*P* < 0.001				
Adhesive-bracket	1 (Control)	0	0	0		0	0	0		*P* = 0.505
	2 (Demineralised/control)	0	0	0	b	0	0	0	b	*P* = 0.718
	3 (Demineralised/NaOCl)	0	0	0		0	0	0	b	*P* = 0.239
	4 (Demineralised/SEP)	0	0	0.44		0	0	0.87		*P* = 0.184
	5 (Demineralised/NaF)	0	0	0	b	0	0	0	b	*P* = 0.336
	Statistical significance	*P* = 0.009				*P* < 0.001				

^a^ statistically significant difference with group 5; ^b^ statistically significant difference with group 4.

### Discussion

The present study investigated the effects of several preparation methods of demineralised enamel on microleakage under metallic orthodontic brackets. The dye penetration with 0.5% basic fuchsine solution was used for microleakage assessment due to its accuracy and cost-effectiveness and to provide results comparable with those of previous studies.

The high prevalence of incipient caries in patients undergoing fixed orthodontic treatment has been an issue of concern for many years, provoking researchers to find treatment modalities to counteract this phenomenon. It is believed that microleakage at the adhesive-bracket interface may cause bracket detachment while microleakage at the enamel-adhesive interface is conductive to the occurrence of white spot lesions [17]. Because hypomineralised enamel is more porous and has less mineral content than sound enamel [25-27], it is possible that microleakage occurs more extensively under brackets bonded to this type of enamel. Microleakage in these teeth may result in more destructive outcomes because hypomineralised enamel is more susceptible to development of caries than sound enamel [7,26,28].

In groups 2 to 4, microleakage occurred predominantly at the enamel-adhesive than at the adhesive-bracket interface, implying that in hypomineralised enamel, microleakage may cause more cases of enamel demineralisation than bracket detachment, a phenomenon that is certainly undesirable. The gingival side generally exhibited higher microleakage value compared with that observed on the occlusal side for both the enamel-adhesive and adhesive-bracket interfaces, but the difference was not statistically significant for any of the study groups. Several studies [6,18,19,22] have reported statistically greater microleakage in the gingival rather than the occlusal margins, ascribing this difference to either the surface curvature anatomy which may result in relatively thicker adhesive at the gingival side [6,19] or to a curing method that applies light purely from the occlusal side [18,22]. To counteract the effect of light direction on microleakage, we applied light from four sides of the bracket.

In this study, hypomineralised teeth prepared by conventional acid etching showed greater microleakage than etched sound enamel at the enamel-adhesive interface of both the occlusal and gingival sides, but the difference was not statistically significant. Pretreatment of hypomineralised enamel with a 5% NaOCl solution did not have a significant effect on microleakage. Several authors [9,10] recommended pretreatment of MIH-affected teeth with 5% NaOCl to improve bonding, but there is no study that has evaluated the effects of deproteinisation on the amount of microleakage under brackets bonded to teeth with hypomineralised defects. There are controversies [11,12,29] regarding the use of NaOCl either before or after acid etching. William et al. [10] recommended initial etching of the hypomineralised defect with 37% phosphoric acid, applying 5% NaOCl and then re-etching the enamel surface before resin placement. It is possible that the latter technique provides better bonding and reduces the amount of microleakage, but further research is required to confirm this assumption.

In restorative dentistry, William et al. [13] were the first to explore the effects of self-etching adhesives on hypomineralised enamel, reporting better bonding of a self-etching adhesive than a total-etching system to this type of enamel. Self-etching primers are also becoming popular in orthodontic treatments because they can simplify orthodontic bonding procedures by decreasing bonding steps and thus reduce chair time. Transbond Plus is an orthodontic self-etching primer with a pH of about 1 that has shown promising results in several studies [30,31]. In this study, preparation of hypomineralised enamel with Transbond Plus self-etching primer caused significantly higher microleakage at the adhesive-bracket interface than when the conventional acid-etching or NaF treatment were used. This finding corroborates the results of Uysal et al. [22] and Hammamci et al. [23] who reported that brackets bonded with self-etching primer revealed significantly higher microleakage at the gingival side compared to that observed with conventional acid etching. However, in the above mentioned studies [22,23], the statistical difference between the microleakage scores of self-etching and conventional acid etching was noted at the enamel-adhesive rather than the adhesive-bracket interface.

In the present study, NaF-treated demineralised enamel revealed the best microleakage resistance, showing statistically lower microleakage values at the enamel-adhesive interface compared to that observed in the other study groups. It has been reported that after acid etching of fluoride-treated caries-like lesions with 20% to 40% unbuffered acid phosphoric solutions, the surface morphology resembled that described for etched sound enamel, although the effect varied with the length of exposure to the etching agent [14-16]. Schimidlin et al. [32] reported that fluoride-treated, acid-etched demineralised enamel allowed good penetration of a bonding agent. The low microleakage scores observed in the hypomineralised/NaF group may be related to adequate resin adhesion and high bond strength, as observed by previous authors [32][33]. However, the statistically lower amount of microleakage in NaF-treated hypomineralised enamel compared to that observed in sound enamel suggests that other factors contribute to reducing microleakage. The precipitation of calcium fluoride on the surface of NaF-treated hypomineralised enamel may have a great inhibitory effect on microleakage. To clarify this assumption, further research is required to determine the amount of microleakage in NaF-treated sound enamel.

The findings of this study revealed that the application of 2% sodium fluoride gel for 4 min before acid etching is a suitable way to reduce the amount of microleakage while promoting re-mineralisation of the underlying lesions in teeth with hypomineralised defects. This may have great clinical implications when one considers the high prevalence and the rapid development of dental caries in patients with hypomineralised enamel [7,26,28]. A limitation of this study was that it determined the short-term microleakage, while in clinical conditions, brackets are generally left in the mouth for approximately 2 years or even more. Further research is required to investigate the effect of enamel treatment with other re-mineralising agents or use of self-etching primers with different acidity on the reduction of microleakage under brackets bonded to hypomineralised enamel.

## Conclusions

The following conclusions were drawn after the experiment: 

1. Brackets bonded to acid-etched demineralised enamel showed higher microleakage than did acid-etched sound enamel at the enamel-adhesive interface on both the occlusal and gingival sides, but the difference was not statistically significant.

2. In demineralised enamel, microleakage occurred mainly at the enamel-adhesive than the adhesive bracket interface, proposing a greater risk of enamel demineralisation upon occurrence of microleakage.

3. Enamel deproteinisation with a 5% sodium hypochlorite solution failed to reduce microleakage under brackets bonded to demineralised enamel.

4. The use of Transbond Plus self-etching primer for preparation of demineralised enamel significantly increased microleakage at the adhesive-bracket interface on both sides of the brackets compared to that observed with conventional acid etching or NaF treatment.

5. The application of 2% sodium fluoride gel for 4 min before acid etching of demineralised enamel significantly reduced microleakage at the enamel-adhesive interface on both sides of the brackets compared to that observed in other study groups.

## Competing interests

The authors declare that they have no competing interests.

## Authors’ contributions

HM (Moosavi) carried out the microleakage assessment. FA supervised the bonding procedure and drafted the manuscript. HM performed the surface treatments and prepared the specimens for microleakage assessment. All authors read and approved the final manuscript.
